# Generation of Internal-Image Functional Aptamers of Okadaic Acid via Magnetic-Bead SELEX

**DOI:** 10.3390/md13127066

**Published:** 2015-12-17

**Authors:** Chao Lin, Zeng-Shan Liu, Dong-Xu Wang, Lin Li, Pan Hu, Sheng Gong, Yan-Song Li, Cheng Cui, Zong-Cheng Wu, Yang Gao, Yu Zhou, Hong-Lin Ren, Shi-Ying Lu

**Affiliations:** 1 Key Laboratory of Zoonosis Research, Ministry of Education, Institute of Zoonosis, Jilin University, Changchun 130062, China; sara851109@hotmail.com (C.L.); zsliu1959@163.com (Z.-S.L.); justme20150701@163.com (L.L.); hupan84@163.com (P.H.); gongsheng1105@126.com (S.G.); l_ys92305@163.com (Y.-S.L.); ccjlucornell@sina.com (C.C.); Wuzongcheng66@126.com (Z.-C.W.); gy497367360@sina.com (Y.G.); zhouyurunye@sina.com (Y.Z.); 2 Jilin Provincial Key Laboratory of Animal Embryo Engineering, College of Animal Science, Jilin University, Changchun 130062, China; wang_dong_xu@163.com; 3 Institute of Wild Economic Animals and Plants, Chinese Academy of Agricultural Sciences, Jilin, Changchun 130062, China

**Keywords:** okadaic acid, SELEX, internal-image function

## Abstract

Okadaic acid (OA) is produced by *Dinophysis* and *Prorocentrum dinoflagellates* and primarily accumulates in bivalves, and this toxin has harmful effects on consumers and operators. In this work, we first report the use of aptamers as novel non-toxic probes capable of binding to a monoclonal antibody against OA (OA-mAb). Aptamers that mimic the OA toxin with high affinity and selectivity were generated by the magnetic bead-assisted systematic evolution of ligands by exponential enrichment (SELEX) strategy. After 12 selection rounds, cloning, sequencing and enzyme-linked immunosorbent assay (ELISA) analysis, four candidate aptamers (O24, O31, O39, O40) were selected that showed high affinity and specificity for OA-mAb. The affinity constants of O24, O31, O39 and O40 were 8.3 × 10^8^ M^−1^, 1.47 × 10^9^ M^−1^, 1.23 × 10^9^ M^−1^ and 1.05 × 10^9^ M^−1^, respectively. Indirect competitive ELISA was employed to determine the internal-image function of the aptamers. The results reveal that O31 has a similar competitive function as free OA toxin, whereas the other three aptamers did not bear the necessary internal-image function. Based on the derivation of the curvilinear equation for OA/O31, the equation that defined the relationship between the OA toxin content and O31 was *Y* = 2.185*X* − 1.78. The IC_50_ of O31 was 3.39 ng·mL^−1^, which was close to the value predicted by the OA ELISA (IC_50_ = 4.4 ng·mL^−1^); the IC_10_ was 0.33 ng·mL^−1^. The above data provides strong evidence that internal-image functional aptamers could be applicable as novel probes in a non-toxic assay.

## 1. Introduction

Okadaic acid (OA), a key toxin involved in diarrhoeic shellfish poisoning (DSP), and is produced by the *Dinophysis* and *Prorocentrum dinoflagellates* [1,2]. This toxin is ingested through a filter feeding mechanism by various species of shellfish, such as bivalve mussels, scallops, oysters, and clams. The toxin does not have a harmful effect on the bivalves [3,4,5]. However, the consumption of contaminated shellfish by humans causes diarrhoeic shellfish poisoning that is characterized by symptoms such as abdominal pain, nausea, vomiting, and diarrhoea [6]. Moreover, OA has also induced carcinogenic, mutagenic, and immunotoxic effects in animal studies [7,8]. Because the contamination of shellfish with OA has harmful impacts on human health, the European Union has limited permissible OA levels to 160 ng·g^−1^ in mussels (EC No. 2074/2005 17) [9]. In China, the maximum permissible dose of OA in seafood is primarily based on the standard of the European Union. Recently, several detection methods for OA have been developed. The mouse bioassay [10] has typically served as the reference method for OA analysis. However, this method has been prohibited since 2011 due to a lack of sensitivity, accuracy and ethical concerns. For this reason, HPLC coupled to various detectors, including mass spectrometry, tandem mass spectrometry (MS/MS), and fluorescence detection are employed to quantify OA and its analogues [11,12,13,14]. The drawbacks of this method are the requirements for highly qualified personnel, enormous sample cleanup, and costly equipment. Recently, immunological analysis methods have been investigated as emerging screening tools for OA because they are rapid, simple and cost effective [15,16,17,18]. Nevertheless, the preparation of the monoclonal antibody is very expensive and time consuming, and the procedure requires the continued use of animals. Moreover, OA toxicity is potentially harmful to the operator’s health.

Aptamers, such as nucleic acid molecules, are selected *in vitro* to bind to molecular targets with high affinity and specificity [19], and also can mimic antigen binding to an antibody in theory. Typically, aptamers are generated through systematic evolution of ligands by exponential enrichment (SELEX), which includes an iterative process of binding, separation, and nucleic acid amplification [20,21]. After multiple rounds of selection, sufficiently specific aptamers are isolated that can selectively bind to peptides, proteins and pathogenic targets by hydrogen bonding, van der Waals forces, hydrophobic effects and other mechanisms [22,23,24,25,26]. The important advantages of aptamers include stability, reduced cost, ease of production, easier incorporation of chemical modifications and batch-to-batch consistency [27]. Over the last few years, the use of aptamers as recognition probes have emerged due to their potential applicability for the determination of marine toxins.

**Figure 1:**
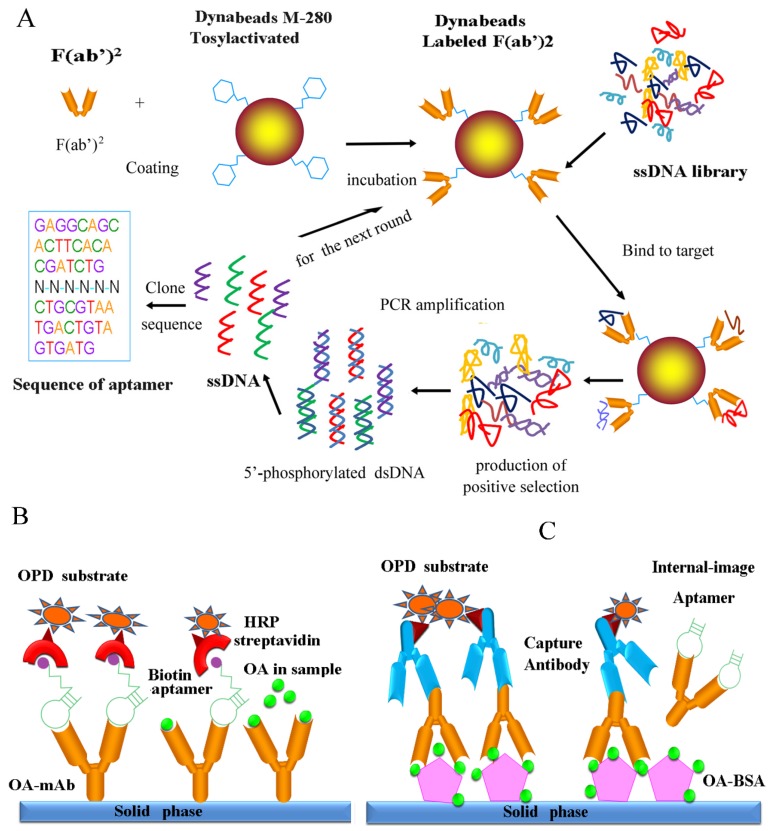
SELEX and Reaction Scheme. (**A**) The SELEX scheme via magnetic beads. First, the F(ab′)^2^ fragment was incubated with tosylactivated dynabeads in binding buffer. To ensure high binding efficiency, the optimal concentration used for the protein and dynabeads was 3 μg F(ab′)^2^ fragment/10^7^ dynabeads. Second, the ssDNA library was added to the pre-coated dynabeads for incubation with the target protein. To ensure highly stringent binding conditions, the ratio of ssDNA and target protein was 1:200. Third, the superparamagnetic beads carrying the target protein and target-bound aptamers were trapped using a magnetic rack. The unbound ssDNA was removed via stringent washing. The bound aptamers were eluted from the beads by heating to 80 °C. The positive aptamers were isolated via magnetic separation. Fourth, the product of selected aptamers was amplified by PCR with a 5′-phosphorylated reverse primer. Next, single-stranded DNA was generated by adding the lambda exonuclease that selectively digests the 5′ phosphorylated strand of the dsDNA. The above four steps were repeated for 10-12 rounds. Finally, the isolated aptamers were cloned and sequenced to obtain candidate aptamers. (**B**,**C**) Two alternative assays for the detection of OA based on aptamers; In (**B**), the biotin-aptamer, as a substitute of OA standard, could limit the binding between the OA-mAb pre-coated and OA toxin in samples; In (**C**), the aptamer, as a substitute of OA standard, could limit the binding between the OA-mAb and the OA-BSA pre-coated in the carrier.

The OA toxin is inevitably used in immunological assays as detection antigen, enzyme conjugate and standard, but is very expensive, and moreover is a potential carcinogenic toxin. The commercial kits for OA (*i.e.*, the Abraxis ELISA kit) are expensive and the use of the toxin as a standard in the assay can be harmful to the operators. Therefore, these kits are not suitable for quantifying contaminants in mass samples, and the development of a surrogate for the OA toxin is urgently required. According to a previous report, the selection of aptamers targeting monoclonal antibodies could be applied in the development of an enzyme immunoassay detecting the small molecule [28]. Therefore, this study aimed to obtain an aptamer specific to the antigen binding site of monoclonal antibody against OA in order to establish a non-toxin or low-toxin assay based on the internal-image aptamer for the detection of OA in seafood. This work is the first report of screening the aptamer capable of binding to the monoclonal antibody against OA as a novel non-toxin probe. Aptamers with high affinity and selectivity that can mimic the OA standard were generated using a magnetic bead-assisted SELEX strategy (Figure 1A). This report represents an alternative probe for development of a non-toxin assay based on using internal-image aptamers for OA as shown in Figure 1B and a low-toxin assay in the Figure 1C, respectively. The biotin-aptamer, as a substitute of OA standard, could limit the binding between the OA-mAb pre-coated on the plate and OA toxin in samples (non-toxin assay; Figure 1B). In the other scheme of low-toxin assay (Figure 1C), the aptamer as a substitute of OA standard could limit the binding between the OA-mAb and OA-BSA pre-coated in the carrier and can be used for the calibration curve.

## 2. Materials and Methods

### 2.1. Reagents and Materials

The IgG antibody against OA used to prepare the F(ab′)^2^ fragments and six IgG antibodies against shellfish toxins were previously generated [16]. OA-BSA was prepared according to the method described in our previous report [16]. The F(ab′)^2^ preparation kit (Pierce) and lambda exonuclease were purchased from Thermo Scientific (Rockford, IL, USA). The synthetic random ssDNA library, primers and biotin-labeled primers listed in Table 1 were synthesized by Shanghai Sangon Biological Engineering Technology & Services Company (Shanghai, China). Bovine serum albumin (BSA) was purchased from Beijing Dingguo Biotechnology Development Centre (Beijing, China). Streptavidin conjugated to horseradish peroxidase was purchased from Bioss Synthesis Biotechnology Co., LTD (Beijing, China). The 96-well ELISA plates were purchased from Costar (Corning, NY, USA). Deionized and pyrogen-free water was obtained from a Milli-Q water purification system (Millipore, Boston, MA, USA). TaqDNA polymerase and PMD-18T were purchased from TaKaRa (Dalian, China). Other reagents were of analytical purity.

**Table 1:** Primer sequences used in this work.

Sequence ID	Sequence (5′-3′)
ssDNA library	5′-GAGGCAGCACTTCACACGATCTG-N40-CTGCGTAATGACTGTAGTGATG-3′
forward primer	5′-GAGGCAGCACTTCACACGAT-3′
reverse primer	5′-CATCACTACAGTCATTACGCAG-3′
biotin-primer	Bio-GAGGCAGCACTTCACACGAT-3′
phosphate-primer	Pho-CATCACTACAGTCATTACGCAG-3′

### 2.2. Preparation of the F(ab′)^2^ Fragment as a Screening Target

F(ab′)^2^ fragments were generated by pepsin digestion of whole IgG antibody against OA with the Pierce F(ab′)^2^ preparation kit according to the manufacturer’s protocols. This digestion removed most of the Fc region while leaving some of the hinge region intact. Complete separation of the F(ab′)^2^ fragment from undigested IgG antibody was assessed using non-reducing SDS-PAGE and Coomassie brilliant blue R-250 staining.

### 2.3. Immobilization of F(ab′)^2^ Fragments Onto Dynabeads

M-280 tosylactivated dynabeads were used as immobilization supports and were handled according to the manufacturer’s protocol. Briefly, the M-280 tosylactivated dynabeads were washed three times with 0.1 M borate buffer (pH 9.5) prior to coating. A total of 1 μmol mAb was added to 300 μL of thoroughly resuspended dynabeads, followed by vortexing for 1 min. After incubation for 20 h at 37 °C with slow tilt rotation, the tubes were placed on a magnet for 2 min, and then the supernatants were removed. Subsequently, the coated beads were washed four times: the first two times in phosphate buffered saline (pH 7.4) with 0.1% (*w*/*v*) BSA for 5 min at 4 °C, once with 0.2 M Tris (pH 8.5) with 0.1% (*w*/*v*) BSA for 4 h at 37 °C to block free groups and one more time in phosphate buffered saline (pH 7.4) with 0.1% (*w*/*v*) BSA for five minutes at 4 °C.

### 2.4. In Vitro Selection of Aptamers for OA-mAb-F(ab′)^2^

For the first round of SELEX, 2 OD of the synthetic ssDNA library in binding buffer (20 mM Hepes, 150 mM NaCl, 2 mM KCl, 2 mM MgCl_2_, and 2 mM CaCl_2_, pH 7.4) was heated at 95 °C for 10 min, rapidly cooled for 5 min in an ice bath, and then incubated for another 5 min at room temperature. Then, 10 mg OA-mAb-coated beads were added and incubated with the library for 2.5 h at room temperature. Nonspecifically bound DNA molecules were removed by washing the OA-mAb-coated beads with binding buffer containing 0.01% Tween-20. OA-mAb-bound ssDNA was eluted at 80 °C for 15 min. Then, the eluted oligonucleotides were purified by ethanol precipitation. To avoid enrichment of nonspecific ssDNAs during the selection process, negative selection with uncoated magnetic beads and six additional mAb (against STX, BTX-2, TTX, DA, NOD and MC-LR)-bead assemblies were used to remove nonspecifically bound ssDNA.

### 2.5. PCR Amplification

A 49 μL PCR mixture containing nuclease-free water, 10 μM forward primer, 10 μM phosphate-labelled reverse primer, 2.5 mM dNTPs and 2.5 U Taq DNA polymerase were prepared. This PCR mixture was combined with 1 μL of the ssDNA collected from the beads. Taq DNA polymerase was activated prior to PCR by heating the reactions to 96 °C for 2 min, followed by 25 cycles of a rapid three-step PCR (30 s denaturation at 96 °C, 30 s annealing at 64 °C, and 30 s extension), followed by a final extension for 5 min at 72 °C.

### 2.6. ssDNA Generation

The ssDNA was prepared as previously described [29]. Briefly, the dsDNA from the PCR product was incubated with the lambda exonuclease for 30 min at 37 °C in 1× Lambda Exonuclease Reaction Buffer to selectively digest the 5′ phosphorylated strand of the dsDNA. The enzyme has reduced activity against ssDNA. The generated ssDNA was purified by ethanol precipitation.

### 2.7. Cloning, Sequencing and Structure Analysis of the Selected Aptamers

After 12 rounds of selection, the selected aptamer pool was amplified by PCR with unlabelled forward and reverse primers. The PCR products were purified by ethanol precipitation and cloned into *Escherichia coli DH5a* using the 18T vector system. Separate colonies were randomly picked and sequenced at Santon Biotech (Shanghai, China) Co., Ltd. Secondary structure analysis was performed with mfold web server.

### 2.8. Affinity Measurements of Selected Aptamers

An indirect biotin-avidin ELISA was developed to measure the affinity of the aptamers referring to the monoclonal antibody affinity measurement [30]. A total of 100 μL of OA-mAb in sodium carbonate buffer was added into each well of a microplate at concentrations of 1, 2, 4, and 8 μg·mL^−1^ and incubated at 4 °C overnight. Subsequently, the plates were blocked with 0.01 M PBS containing 5% skim milk powder for 2 h at 37 °C. Synthetic biotin-aptamers pre-denatured for 10 min at 96 °C and immediately cooled on ice in 2× binding buffer were added (100 μL per well). After incubation for 45 min at 37 °C, unbound molecules were washed away with 1× binding buffer containing 0.01% Tween-20. Next, HRP-streptavidin at a 1:1000 dilution was added to bind biotin for 30 min at 37 °C. Lastly, the o-phenylenediamine dihydrochloride (OPD) substrate system was employed to measure the OD_492_ values using a microplate reader from BioTex (Odense, Denmark).

An indirect competitive ELISA (ic-ELISA) was used to determine the IC_50_ and IC_10_ values of the candidate aptamers. The procedure was performed as follows: the OA-BSA conjugate was immobilized in a microtiter plate as the detection antigen. Next, the OA standard or aptamers with gradient dilutions were added into the wells. The antibody OA-mAb was immediately added at a 20,000-fold dilution. After incubation at 37 °C for 1 h, goat anti-mouse IgG antibody labelled with HRP was added to the microplate as the capture antibody to bind to the Fc fragment of OA-mAb. The *O*-phenylenediamine substrate system was used to measure the OD_492_ values. Analysis of the OD_492_ values were undertaken to determine the relative relationship between OA and the selected aptamers.

### 2.9. Identification and Characterization of Internal-Image Aptamers

An ic-ELISA was employed to identify the internal-image function of O31 screened via magnetic-bead SELEX. After biosynthesis of O31 by Shanghai Sangon Biological Engineering Technology & Services Company, the procedure was performed in accordance with the steps pre-described in Section 2.8.

### 2.10. Inter and Intra-Assay Variation

The inter and intra-assay variation of the selected aptamers were also evaluated. Based on the value determined using the calibration curve, 0, 10, or 50 ng·mL^−1^ OA solutions were chosen to test the inter and intra-assay variation. The experiments performed for the analysis were repeated three times a day for three days.

## 3. Results and Discussion

### 3.1. Preparation of F(ab′)^2^ Fragments

To improve the efficiency of SELEX, the F(ab′)^2^ fragment was considered to be the screening target able to maintain competent the antigen binding sites. F(ab′)^2^ fragments have the advantage of a small molecular weight and reduced steric hindrance due to the lack of most of the Fc region. Therefore, the use of F(ab′)^2^ fragments as a screening target could reduce the probability of producing non-specific aptamers. OA-mAb was digested with pepsin using the F(ab′)^2^ preparation kit (Pierce). Next, native-PAGE was used to identify the F(ab′)^2^ fragment. The molecular weight of the F(ab′)^2^ fragment of the OA-mAb was between 97 kD and 160 kD. Therefore, the preparation of the F(ab′)^2^ fragments (110 kD) was considered to be successful. Additionally, high purity of the F(ab′)^2^ fragments, as verified by electrophoresis were achieved; thus, the fragments could be used in the following experiments.

### 3.2. Immobilization of OA-mAb-F(ab′)^2^ onto Dynabeads

**Figure 2:**
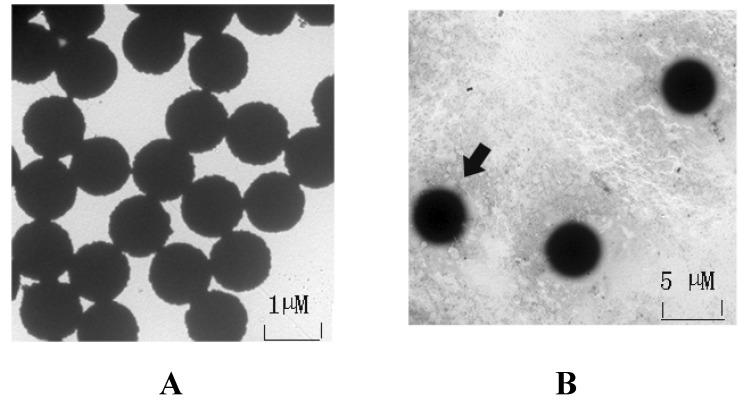
Transmission electron microscopy of the superparamagnetic beads. (**A**) Transmission electron microscopy of tosylactivated dynabeads. In the scale of the TEM, the magnetic beads were uniformly dispersed and possessed of a consistent size; **(B**) Transmission electron microscopy of beads coated with F(ab′)^2^ fragments. The superparamagnetic beads carrying the target protein were verified by transmission electron microscopy. In (**B**), a “protein cloud” can be observed around the superparamagnetic beads. The F(ab′)^2^ fragment was stained with phosphotungstic acid; thus, the protein on the face of the magnetic beads was dark.

The OA-mAb-F(ab′)^2^ was covalently coupled onto the surface of tosylactivated dynabeads using the primary amino groups (NH_2_) of the antibody. The coupled complex was observed by transmission electron microscopy (TEM, H-7650). The images revealed that the uncoupled magnetic beads were homogeneously distributed in the field of view. Importantly, the OA-mAb-F(ab′)^2^-bead assemblies were encircled by a protein halo, indicating that OA-mAb-F(ab′)^2^ were successful coated onto the magnetic beads (Figure 2A). The coupling ratios were calculated by determining the concentration of OA-mAb-F(ab′)^2^ in the supernatant. The difference in the concentrations of the overall protein and the un-reacted protein divided by the concentration of the overall protein was approximately 85%. Based on the above data, we can conclude that OA-mAb-F(ab′)^2^ was successfully immobilized onto the face of the magnetic beads.

### 3.3. In Vitro Selection of OA-mAb-F(ab′)^2^-Targeting Aptamers

The SELEX process began with the incubation of 2 nmol of the random ssDNA library (>10^14^ molecules) with target proteins conjugated to magnetic beads. To ensure highly stringent selection conditions, the molar ratio between the ssDNA and target protein was maintained at >100:1 as described previously [31]. After the first round of SELEX, the ssDNA pool was amplified by PCR. The annealing temperature is a major factor that affects the specificity of PCR products. So to avoid nonspecific amplification, gradient temperature PCR was chosen as the optimization method. The result showed that 63 °C was optimal for the production of a high content of dsDNA with sufficient purity (data not shown).

The generation of high purity ssDNA is a key factor to obtain aptamers with high affinity and selectivity. In this study, lambda exonuclease was used to generate ssDNA with a 5′ phosphorylated reverse primer. The phosphorylated dsDNA strand produced by PCR was digested into pieces by the lambda exonuclease. The reaction was stopped by heating at 80 °C for 15 min, followed by ethanol precipitation. In contrast to the traditional preparation of ssDNA using asymmetric PCR, enzyme digestion resulted in the generation of higher purity ssDNA, which plays an important role in the enrichment of specific aptamers. Additionally, this method for the generation of ssDNA as a library for the next round of SELEX saves time, and required just 25 min. To avoid the production of nonspecific aptamers, a high annealing temperature was imposed during the PCR amplification in addition to the stringent washing conditions (Table 2).

The products from the previous round of selection after the generation of ssDNA were used as the secondary pool for the next round of selection. Although the use of a rich ssDNA library with a variety of secondary structure could increase the probability of the selection of specific aptamers, this strategy could also result in a drop in efficiency. Therefore, the ssDNA contents of the secondary pool should be reduced to 1 nmol. Following the increasing selection rounds, the pool contents were gradually decreased from 2 nmol to 0.1 nmol to improve the SELEX efficiency. The stringent washing condition used for SELEX ensured the rapid selection of high affinity and specificity sequences for the target protein.

**Table 2:** The SELEX conditions.

SELEX	ssDNA Library	Target	Elution Times
Rounds	(pmol)	(μM)	(times)
1	2000	200	6
2	1500	200	9
3	1000	150	10
4	500	150	10
5	500	100	15
6	400	100	15
7	200	50	18
8	200	50	20
9	200	25	20
10	100	25	25
11	100	20	25
12	100	10	25

### 3.4. The Result of SELEX

**Figure 3:**
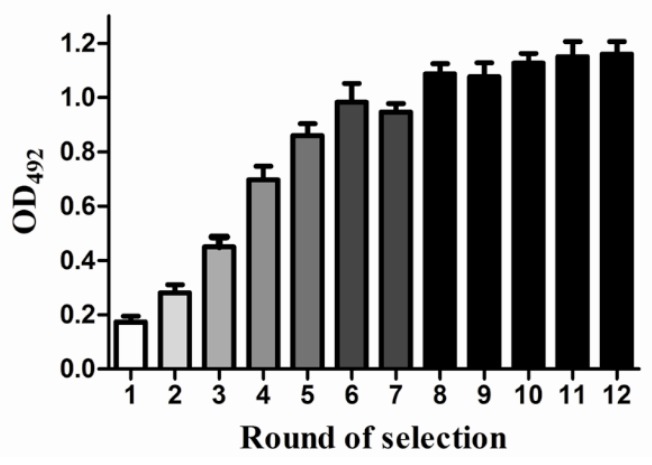
The rounds of SELEX. A biotin-avidin ELISA was used to test the OD values of rounds of selection using the ssDNA pool. The OD value of each round was expressed as the mean ± SD (*n* = 3). Counter selection was performed using other related antibody-coated magnetic beads after rounds five and seven. After the 12th round of selection, the ssDNA library enrichment had reached saturation.

A biotin-avidin ELISA was used to monitor the enrichment. As shown in Figure 3, the OD values progressively increased from round 1 to round 11. The significant increasing OD value demonstrated that the ssDNA binding to the target protein was enriched. However, there was no obvious difference between round 11 and round 12. Moreover, there was no significant increase in the OD value after round 12 (data not shown), indicating that the assay reached its plateau and that the binding affinity was saturated. The OD value in the seventh SELEX rounds decreased modestly compared to the OD of round six, most likely due to the presence of nonspecific aptamers in the ssDNA pool. The nonspecific aptamers interfere with the binding between the specific ssDNA and the target protein therefore causing the lower OD value. This result indicated that the nonspecific aptamers were being discarded from the ssDNA library.

### 3.5. Prediction of the Secondary Structures of Selected Aptamers

**Figure 4:**
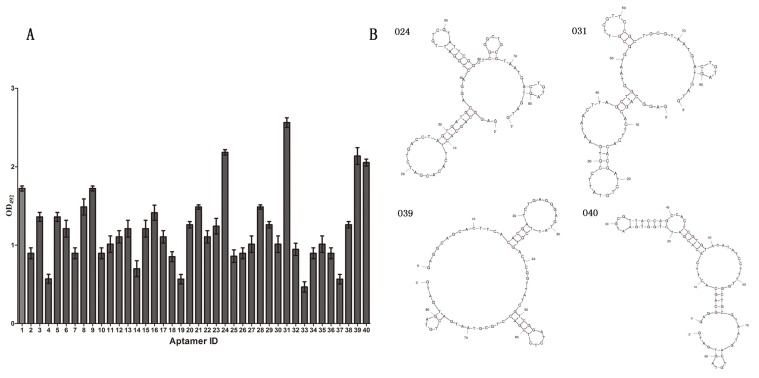
(**A**) The affinity of random clones. The OD values of the forty cloned sequences were detected using indirect ELISA. O24, O31, O39 and O40 were chosen as candidate aptamers because of their higher affinity; (**B**) Predicted secondary structures of four candidate aptamers. Predicted secondary structures of aptamers O24, O31, O39 and O40 determined by the mfold tool.

Forty clones were randomly chosen after 12 rounds of selection. The affinity of the forty clone sequences was assessed by indirect ELISA. As shown in Figure 4A, the OD values of O24, O31, O39 and O40 aptamers were significantly higher than those of the other aptamers. Therefore they were considered suitable candidates for further investigation. The mfold program was used to predict their secondary structures of the four candidate aptamers according to the principle of minimum energy. As shown in Figure 4B, the four candidate aptamers folded into three or four stem-loop structures and the loops in each of the aptamers corresponded to the random region. A few stem-loop structures ensure the three-dimensional conformation of aptamers that could bind to the antibody. The plentiful spatial arrangement was important in binding to antibody. The enrichment of GC sequences in the hairpins of four candidate aptamers ensured that their structures were stable. Importantly, the hairpins may directly participate in the binding between the aptamers and the monoclonal antibodies.

### 3.6. The Results of the Specificity and Ka Tests

**Figure 5:**
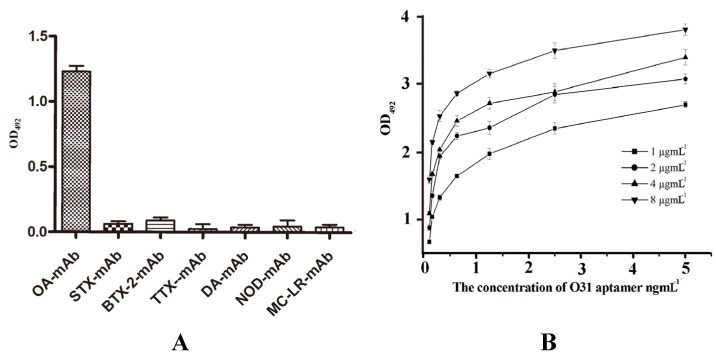
Characterization of the O31 aptamer. (**A**) The specificity of the O31 aptamer. The six monoclonal antibodies against the marine toxin (as prepared previously by our lab [32,33,34,35]), were used to test the specificity of O31. The OA-mAb and six other monoclonal antibodies were coated on magnetic beads. Then, the proper dilution of the O31 aptamer was added to the tubes. The OD_492_ values were measured to analyse the specificity; (**B**) Affinity constants value for the O31 aptamer. The synthetic biotin-aptamers were added at concentrations of 0.1, 0.155, 0.31, 0.625, 1.25, 2.5 and 5 ng·mL^−1^ to 1, 2, 4, and 8 μg·mL^−1^ of OA-mAb pre-coated on the plate.

O24, O31, O39 and O40 were selected to determine the specificity of the aptamers. The six monoclonal antibodies against OA, STX, BTX-2, TTX, DA, NOD and MC-LR stored in our lab were coated onto the faces of magnetic beads. The other steps were in keeping with the indirect ELISA as described in Section 2.8. The results obtained using O31 are shown in Figure 5A. O31 exclusively bound to OA-mAb, and did not exhibit any cross-reactivity with the other monoclonal antibodies. Similar results were obtained for O24, O39 and O40. To test the affinity of the individual aptamers for their target, the OD values were determined by ELISA. The aptamers showed a high affinity for mAb-OA. As shown in Figure 5B, different concentrations of aptamers were tested to determine their OD values. The binding affinity of the aptamer was calculated using the following equation: *K*a = (*n* − 1)/(*n*[Ap′]*t* − [Ap]*t*). In this equation, n is the concentration ratio of plates coated with two different concentrations of F(ab′)^2^ in one group and [Ap′] and [Ap] are the concentrations (mol L^−1^) of the aptamers corresponding to 50% of the maximum absorbance values of the plates coated with the two different concentrations of OA-BSA. The average was the affinity constant. The affinity constants of O24, O31, O39 and O40 were determined to be 8.3 × 10^8^ M^−1^, 1.47 × 10^9^ M^−1^, 1.23 × 10^9^ M^−1^ and 1.05 × 10^9^ M^−1^, respectively. The data illustrated that the four aptamers had binding capacities similar to the binding capacity of OA-mAb. Because the O31-mAb compound had a relatively slow dissociation process, it was considered to have a slightly stronger binding capacity. In contrast, O24 showed a relatively low affinity with OA-mAb. The data described above further demonstrates that the four candidate aptamers have a high affinity and specificity for the OA-mAb.

### 3.7. Internal-Image Function of the Selected Aptamers

**Figure 6:**
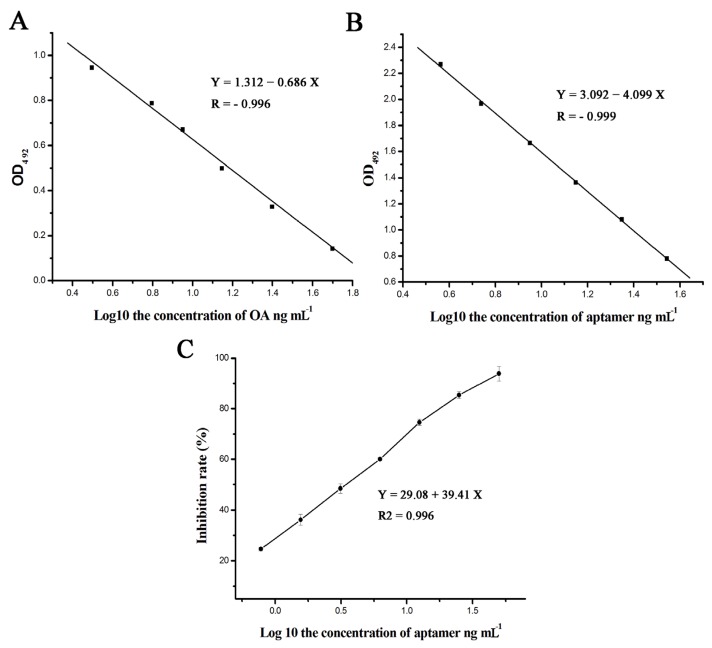
(**A**) The calibration curves for OA by direct competitive ELISA (dc-ELISA). The calibration curves for OA were developed using different dilutions of free OA toxin. The regression equation was *Y* = 1.312 − 0.686*X*; (**B**) The calibration curves for O31 by dc-ELISA. The calibration curves for O31 were developed using different dilutions of biosynthetic aptamer. The regression equation was *Y* = 3.092 − 4.099*X*. The relationship equation between OA and O31 was *Y* = 2.185*X* − 1.78, where *Y* represents the aptamer content and *X* represents the OA content; (**C**) The determination of the O31 IC_50_ value. The regression equation was *Y* = 29.08 + 39.41*X*. The 50% inhibition concentration (IC_50_) of O31 was 3.39 ng·mL^−1^ and the IC_10_ was 0.33 ng·mL^−1^.

The aptamers that mimicked antigen binding to the antibody were used to develop the ELISA. To determine the internal-image function of the selected aptamers, OA and the four aptamers were separately added into microplates pre-coated with OA-BSA. The results indicated that O31 could inhibit OA-mAb as shown in Figure 6A. Interestingly, a similar inhibition ratio was displayed by O31 as an alternate for OA in an ic-ELISA (Figure 6B). Thus, O31 could bind to the OA-mAb as well as recognize the antigenic determinant on the face of the antibody. Furthermore, the binding of aptamer and OA-mAb caused a drop in the binding probability between the antibody and solid phase antigen in ic-ELISA. However, the other three aptamers exhibited only a weak capacity to interfere with the formation of the OA-mAb and OA-BSA compound (data not shown). This result revealed that while O24, O39 and O40 possessed a strong affinity to OA-mAb similar to O31, they did not present the internal-image function because they could not compete for the antibody with the solid phase antigen. The difference in the functional characteristics of the four candidate aptamers could be related to the different sequences and their three-dimensional structures. Taken together, the results demonstrated that only O31 had internal-image functions corresponding to the free OA toxin. Based on the derivation of the curvilinear equation for OA or the aptamer, the equation concerning the relationship between the OA toxin content and the aptamer was *Y* = 2.185*X* − 1.78, where *Y* represents the aptamer content and *X* represents the OA content.

An ic-ELISA was implemented for analyzing thorough advantage of O31 binding to OA-mAb. The competition curves of O31 are shown in Figure 6C; all of the curves exhibited good linearity (*R*^2^ > 0.99). The 50% inhibition concentration (IC_50_) of O31 was 3.39 ng mL^−1^, which was close to the value reported by ELISA (4.4 ng mL^−1^) [16]. The IC_10_ (10% inhibition concentration) value for O31 was 0.33 ng·mL^−1^.

### 3.8. Intra and Inter-Assay Variation

A series of standard solutions (0, 10 and 50 ng mL^−1^) were measured to determine the accuracy and precision of O31 (Table 3). Intra-assay variation was obtained based on triplicate measurements in one day, while the inter-assay variation was calculated based on the results obtained over three days. The results showed that the inter-assay variation (<7%) and the intra-assay variation (<9%) were both acceptable. This finding suggested that the selected aptamer was stable and credible for determining the concentration of OA. This, together with the internal-image function of O31, means that it could serve as a possible replacement for the OA standard in the detection of OA in shellfish.

**Table 3:** Intra-assay and inter-assay variation coefficients.

Apatmer	Concentration (ng·mL^−1^)	Inter-Assay (*n* = 3)		Intra-Assay (*n* = 3)	
		OD_492_ Mean ± SD (X ± SD)	CV%	OD_492_ Mean ± SD (X ± SD)	CV%
Ab-24	0	1.04 ± 0.016	1.5	1.17 ± 0.047	4.0
	10	0.63 ± 0.028	4.4	0.75 ± 0.035	4.6
	50	0.17 ± 0.012	7.0	0.23 ± 0.017	7.4
Ab-31	0	1.12 ± 0.031	2.7	1.02 ± 0.032	3.1
	10	0.62 ± 0.025	4.0	0.55 ± 0.012	2.2
	50	0.09 ± 0.005	5.5	0.08 ± 0.007	8.8
Ab-39	0	1.13 ± 0.032	2.8	1.09 ± 0.023	2.1
	10	0.71 ± 0.016	2.2	0.64 ± 0.015	2.3
	50	0.13 ± 0.009	6.9	0.13 ± 0.002	1.5
Ab-40	0	1.12 ± 0.025	2.2	1.18 ± 0.045	4.4
	10	0.69 ± 0.014	2.0	0.72 ± 0.017	2.4
	50	0.18 ± 0.007	3.8	0.22 ± 0.016	7.2

## 4. Conclusions

In this study, we report for the first time the development of aptamers that can mimic the OA toxin. The four different aptamers, selected based on their affinity for the OA-mAb via magnetic-bead SELEX, were investigated by ic-ELISA. Of the four aptamers, O31 had the highest affinity constant. The ic-ELISA result revealed that only O31 had a similar function with the free OA toxin, which reacted with OA-mAb and reduced the binding probability of OA-BSA and the antibody. The O31 selected in this study showed the best IC_50_ value (3.39 ng·mL^−1^) and had wide applicability for the development of non-toxic detection assays as an alternative for OA. Further work is aimed at using the internal-image functional aptamers to develop a novel assay to detect OA in shellfish.
